# Computed tomographic features of presumed normal palatine tonsils in 140 dogs

**DOI:** 10.1111/jsap.70132

**Published:** 2026-04-12

**Authors:** N. Leong, R. Drees, H. Dirrig

**Affiliations:** ^1^ The Royal Veterinary College Hatfield UK; ^2^ MIRA! Veterinary Diagnostic Imaging LTD Sawston UK; ^3^ Department of Clinical Science and Services, Queen Mother Hospital for Animals The Royal Veterinary College Hatfield UK

## Abstract

**Objectives:**

The purpose of this retrospective study was to describe the CT features of presumed normal canine palatine tonsils.

**Materials and Methods:**

Pre‐ and post‐contrast head CT studies of 140 dogs without tonsillar disease were included. Subjective evaluation of palatine tonsils included contrast enhancement pattern, differentiation from surrounding soft tissues and presence of gas or mineral within the tonsillar fossae. Quantitative measurements included pre‐ and post‐contrast attenuation, width, height and length. Relationships between palatine tonsillar size and age, bodyweight, brachycephalic *versus* normocephalic conformation and concurrent regional disease were investigated. Bodyweight‐based ranges (≤10.0 kg, 10.1 to 25.0 kg and >25.0 kg) for tonsillar sizes were established to assess differences in tonsillar size across weight categories.

**Results:**

Palatine tonsils were identified on CT examination in all dogs. 52.9% palatine tonsils were poorly differentiated from adjacent soft tissues in post‐contrast series, 86.1% tonsillar fossae contained gas and 7.9% contained mineral. Mean palatine tonsillar width was 6.9 mm (SD 2.2 mm), height 6.5 mm (SD 2.0 mm) and length 19.1 mm (SD 4.5 mm). Median attenuation pre‐contrast was 50.8 HU (range 22.6 to 82.1 HU), significantly increasing to 89.4 HU post‐contrast (range 49.7 to 128.0 HU). Tonsillar size was negatively correlated with age and positively correlated with bodyweight. There were statistically significant increases in tonsillar size with each ascending bodyweight range. No statistically significant size differences were found between brachycephalic and normocephalic breeds, nor between dogs with or without concurrent regional disease.

**Clinical Significance:**

These findings may serve as informative references for evaluating palatine tonsils on CT examinations in healthy dogs and provide comparisons for assessing tonsillar pathology.

## INTRODUCTION

Evaluation of canine palatine tonsils during oral examination and in diagnostic imaging studies poses a challenge because of their anatomic location, possible individual variation, and similarity to adjacent oropharyngeal soft tissues (Evans & Lahunta, 2013). As secondary lymphoid organs, the tonsils play an important role in immune surveillance at their position at the intersection of the digestive and respiratory tracts (Casteleyn et al., 2011). The tonsils are comprised of aggregations of specialised lymphoid cells that form mucosa‐associated lymphoid tissue (MALT), similar to the lymphoid tissue found in lymph nodes (Uzal et al., 2016). There are three types of tonsils recognised in the dog: a small and diffuse lingual tonsil located at the base of the tongue, a flattened and diffuse pharyngeal tonsil located in the nasopharynx, and distinct paired palatine tonsils located in the dorsolateral walls of the oropharynx (Casteleyn et al., 2011). The palatine tonsils are ovoid structures that reside within a tonsillar fossa, and consist of MALT surrounded by a capsule (König et al., 2020). They can be further divided into the main fusiform portion, which protrudes into the oropharynx, as well as the deeper and smaller portion that lies within the mucosa in the tonsillar fossa (Evans & Lahunta, 2013). The palatine tonsils receive blood supply from the tonsillar arteries, derived from the lingual arteries and only have efferent lymphatics draining into the medial retropharyngeal lymph nodes (Evans & Lahunta, 2013). Diseases affecting palatine tonsils in dogs include inflammation (relating to infection, allergic reaction or foreign body), neoplasia (most commonly squamous cell carcinoma, lymphoma and melanoma), polyps and cysts (Boot et al., 2024; Carozzi et al., 2015; König et al., 2020; Mickelson et al., 2020; Molín et al., 2021; Uzal et al., 2016). In brachycephalic dogs, the palatine tonsils may be abnormally positioned and/or everted due to breed conformation or secondary to increased negative pressure from brachycephalic obstructive airway syndrome (BOAS) (Ekenstedt et al., 2020; Fasanella et al., 2011; Köhler et al., 2021).

In veterinary practice, visual examination of the palatine tonsils is often challenging unless there are gross abnormalities or obvious enlargement. Non‐invasive imaging is therefore a valuable next step in their evaluation. CT and MRI are commonly used to evaluate the head and neck, enabling assessment of complex anatomical structures due to their cross‐sectional nature, such as the oropharynx (D'Anjou, 2013; Wippold, 2007). However, identifying the palatine tonsils on CT examinations can be difficult due to their isoattenuation with surrounding soft tissues, leading to their potential oversight during CT assessment (Murakami et al., 2018). Previous studies have described neoplastic features of canine palatine tonsils on CT examinations and explored techniques such as barium‐painted contouring of the palatine tonsils for radiotherapy planning (Carozzi et al., 2015; Murakami et al., 2018; Thierry et al., 2018). However, the CT appearance of normal palatine tonsils remains unreported and no reference values have been established, to the authors' knowledge, based on a search of the veterinary imaging literature. In contrast, the MRI appearance of normal canine palatine tonsils has been successfully described (Köhler et al., 2021; Ruiz‐Drebing et al., 2019). Although MRI is generally considered superior for soft tissue contrast, CT is typically more accessible in practice due to its speed, lower cost and utility for assessing other head structures (Forrest & Saunders, 2011; Wippold, 2007). In addition, given that pharyngeal neoplasia in dogs is most commonly found in the tonsillar fossa, a thorough CT evaluation of the head should include assessment of the palatine tonsils, even if they are not the primary focus (Carozzi et al., 2015).

The objective of this study was therefore to describe the features of presumed normal canine palatine tonsils on CT examination. It was hypothesised that pre‐ and post‐contrast CT images of the canine head would (1) allow identification of the palatine tonsils; (2) facilitate subjective assessment of palatine tonsil contrast enhancement pattern, post‐contrast differentiation from surrounding tissue and gas and/or mineral contents in the fossa; (3) enable measurement of palatine tonsil pre‐ and post‐contrast attenuation and width, height and length; and (4) that palatine tonsil size measurements would vary by age, bodyweight, brachycephalic conformation and presence of concurrent regional disease. The results could provide informative reference data for evaluating the palatine tonsils in dogs on CT examinations, helping to distinguish between normal and abnormal palatine tonsil appearances and guiding further patient management. This data may also be useful for comparative purposes in future research on the CT appearance of canine palatine tonsillar disease.

## MATERIALS AND METHODS

### Study design and inclusion criteria

Medical records of 200 dogs that underwent CT examination of the head at the Queen Mother Hospital for Animals between March and November 2023 were reviewed retrospectively. Dogs were included based on the confirmed use of contrast for CT soft tissue imaging of the head. Dogs were excluded if they had a clinical history or imaging findings suggestive of primary tonsillar neoplasia, tonsillar metastasis, tonsillar inflammation, everted/hypertrophic tonsils associated with BOAS, or other tonsillar pathologies. Additionally, dogs with visibly compressed palatine tonsils due to mass lesions or an endotracheal tube, those with palatine tonsils affected by imaging artefacts, or those with scans of inadequate quality were also excluded. If a dog presented multiple times within the study period, only the most recent imaging study was included.

### CT acquisition technique

CT images were acquired using an Aquilion ONE Genesis Edition (320 slice, Canon Medical Systems, Otawara, Japan) CT Unit. Dogs were positioned in sternal recumbency and restrained with either sedation or general anaesthesia. Pre‐ and post‐contrast studies were obtained for each dog, with post‐contrast images acquired 60 to 90 seconds after intravenous contrast administration with iohexol (300 mg/mL, Omnipaque, GE Healthcare AS, Nydalen, Oslo, Norway) at 2 mL/kg through a cephalic vein with a single or dual injector. The following scan parameters were used: helical acquisition, reconstruction slice thickness from 1.0 to 3.0 mm, 120 kVp, mAs individually modulated using SUREExposure™ software (Canon Medical Systems), 0.8 to 1.4 pitch, image field‐of‐view tailored individually, medium frequency spatial reconstruction algorithm. The CT images were reviewed and analysed using eUnity Version 7.3.2‐234 (Mach7 Technologies Canada Inc., Ontario, Canada) online DICOM viewing platform. All images were viewed in a soft tissue window (width 400 Hounsfield Units – HU; level 40 HU), and window width and window level were optimised by the observers if necessary.

### Patient data and image evaluation

The signalment of each included dog was recorded, including their age, sex, neutering status, bodyweight and breed. A summary of the medical history and CT report for each dog was also recorded. Each palatine tonsil was identified and subjectively evaluated in the pre‐contrast series for the presence of gas and/or mineral attenuation within the tonsillar fossae. Gas attenuation was defined as between −1000 and −800 HU, and mineral attenuation was defined as distinct, hyperattenuating foci of ≥100 HU. The contrast enhancement pattern of each palatine tonsil in the post‐contrast transverse series was classified as either heterogeneous or homogeneous. The differentiation of each palatine tonsil compared to surrounding soft tissues was subjectively evaluated in the post‐contrast transverse series, and the degree of differentiation was classified as poor, medium or good. The relationship between brachycephalic conformation and differentiation was assessed.

**FIG 1:**
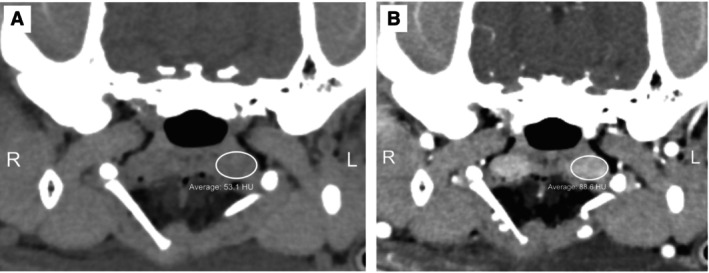
Transverse plane images of the palatine tonsils: an ellipse is placed over the left palatine tonsil to facilitate attenuation measurement: (A) pre‐contrast; (B) post‐contrast.

For objective attenuation measurements, palatine tonsils were evaluated in the slice which contained the largest cross‐section of the palatine tonsil. Attenuation of the palatine tonsils was measured in HU in both pre‐ and post‐contrast images, by placing an electronic oval ellipse region of interest over the palatine tonsil and recording the mean HU value, as shown in Fig 1. The ellipse region of interest was maximised over each palatine tonsil and maintained in the same location in pre‐ and post‐contrast images, ensuring measurement consistency and exclusion of gas or mineral content. Mean post‐contrast attenuation measurements of the jugular vein and common carotid artery were performed by placing an ellipse in the right‐sided vessels approximately at the level of C2. These values were assessed in relation to the palatine tonsil post‐contrast attenuation means, as well as the palatine tonsil contrast enhancement pattern and the degree of differentiation of palatine tonsils from surrounding soft tissues in post‐contrast images.

**FIG 2:**
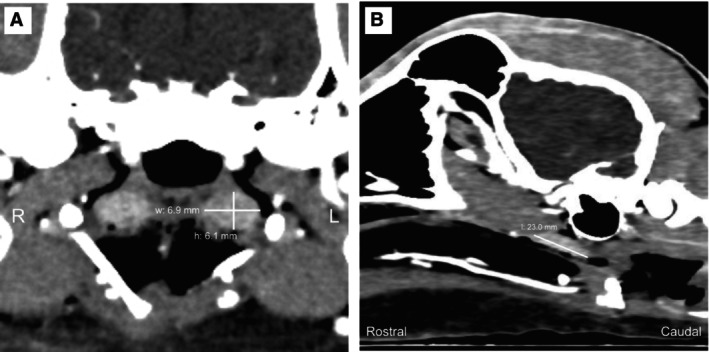
Post‐contrast images of the palatine tonsils: (A) linear callipers are placed over the left palatine tonsil to facilitate width (w) and height (h) measurements in the transverse plane; (B) a linear calliper is placed over the left palatine tonsil to facilitate length (l) measurement in the sagittal plane.

For size measurements, only palatine tonsils exhibiting medium and good differentiation from surrounding soft tissues in post‐contrast images were assessed. Multiplanar reconstruction (MPR) was performed, with the dorsal plane aligned to the hard palate. Dimensions were measured using electronic callipers in the post‐contrast series: width, defined as the maximum linear distance from the medial to lateral border of the palatine tonsil in the transverse plane; height, defined as the maximum linear distance from the dorsal to ventral border of the palatine tonsil in the transverse plane; and length, defined as the maximum linear distance from the rostral to caudal border of the palatine tonsil in the sagittal plane. The size measurement process is shown in Fig 2. Size measurement relationships with age, bodyweight, brachycephalic conformation and concurrent regional disease were assessed. The presence of concurrent regional disease (including head, neck and dental disease) was determined based on the animal's recorded medical history and CT report.

The right and left tonsils were compared with respect to the presence of gas and mineral attenuation within the tonsillar fossae in pre‐contrast images, and tonsillar size (width, height and length), mean pre‐ and post‐contrast attenuation, contrast enhancement pattern and differentiation from surrounding soft tissues in post‐contrast images.

Image review was initially performed by a single reviewer, a final‐year veterinary student. A consensus review with a board‐certified radiologist was achieved for every case for subjective attenuation values (contrast enhancement pattern and degree of differentiation), length/width/height measurements of the palatine tonsils, and the presence of mineralisation and gas within the tonsillar fossae. Vascular attenuation measurements were performed by another board‐certified radiologist.

### Statistical analysis

Statistical analysis was performed with SPSS Version 29.0.2.0 for Macintosh (IBM SPSS Inc., New York, United States of America) statistical software package. Kolmogorov–Smirnov tests with Lilliefors correction were used to assess the normality of the quantitative datasets. Parametric tests and descriptive statistics (mean and standard deviation (SD)) were applied to normally distributed datasets, while nonparametric tests and descriptive statistics (median and range) were used for datasets that did not follow a normal distribution. Chi‐square tests were applied to categorical datasets. Spearman correlation tests were used to evaluate the relationships of palatine tonsillar size with age and bodyweight. Dogs were separated into three bodyweight‐based reference groups (≤10.0 kg, 10.1 to 25.0 kg and >25.0 kg), and an analysis of variance (ANOVA) test was performed to compare palatine tonsillar sizes across these groups. Two‐tailed paired *t*‐tests were used to compare the right and left tonsillar sizes, and Wilcoxon signed‐rank tests were used to compare the right and left pre‐ and post‐contrast attenuation means. Two‐tailed *t*‐tests were used to evaluate the association between palatine tonsillar size and brachycephalic conformation (brachycephalic *vs*. normocephalic dogs), as well as palatine tonsillar size and the presence of concurrent regional disease. A significance level of *P* < 0.01 was used for all tests.

## RESULTS

### Patient findings

A total of 140 dogs matched the inclusion criteria and were included in the study; 60 dogs were excluded. Thirty dogs were of mixed breed. The other dogs represented 50 different breeds: cocker spaniel (*n* = 9), Labrador retriever (*n* = 9), French Bulldog (*n* = 8), springer spaniel (*n* = 8), golden retriever (*n* = 5), Staffordshire Bull terrier (*n* = 5) and Bulldog (*n* = 4). The remaining 43 breeds are listed in online Appendix Table S1. The dogs were further divided into a brachycephalic group (*n* = 26; including boxer, Bulldog, Cavalier King Charles Spaniel, Chihuahua, French Bulldog, Lhasa apso, Pug, shih‐tzu, Staffordshire Bull terrier, Tibetan spaniel) and a normocephalic group (*n* = 114; including all other breeds).

Of the included dogs, 58 were female (42 neutered) and 82 were male (52 neutered). Age and bodyweight were not normally distributed. The median age was 6 years (range 6 months to 16 years) and the median bodyweight was 15.7 kg (range 1.8 to 51.0 kg).

Eighty‐six dogs were scanned under sedation and 54 dogs were scanned under general anaesthesia with an endotracheal tube in place.

Concurrent regional disease was reported in 109 dogs, including dental (*n* = 41), facial, sinonasal, orbital, aural, salivary gland, cervical, musculoskeletal and intracranial pathologies, with aetiologies encompassing inflammatory infectious, inflammatory immune‐mediated, traumatic, neoplastic, anomalous and degenerative processes. Thirty‐one dogs had no reported concurrent regional disease.

### CT findings

A slice thickness of 2.0 mm was used to reconstruct 135 of the CT scans, with fewer scans using a slice thickness of 1.0 mm (*n* = 3) and 3.0 mm (*n* = 2).

#### Palatine tonsil identification

**FIG 3:**
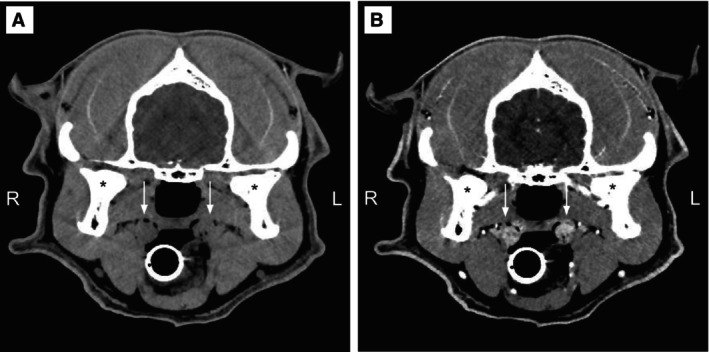
Normal location of the palatine tonsils (arrows) at the level of the temporomandibular joints (asterisks) in the transverse plane: (A) pre‐contrast exam; (B) post‐contrast exam.

The palatine tonsils were successfully identified in all dogs in both pre‐ and post‐contrast images in the soft tissue window. They were consistently located at the level of the temporomandibular joints (TMJs) in the transverse plane, as shown in Fig 3.

#### Right *versus* left palatine tonsils

The right (*n* = 140) and left (*n* = 140) palatine tonsils were compared. No statistically significant differences were found between right and left palatine contrast enhancement patterns (*P* = 1.000), differentiation from adjacent tissues (*P* = .93) and gas contents (*P* = .23) or mineral contents (*P* = .66) of the fossae. Pre‐ and post‐contrast attenuation means were not normally distributed for right and left palatine tonsils, and no statistically significant differences were found between right and left pre‐contrast attenuation means (*P* = .71) and post‐contrast attenuation means (*P* = .93). Widths, lengths and heights were all normally distributed for right and left palatine tonsils, and no statistically significant differences were found between right and left widths (*P* = .41), heights (*P* = .86) and lengths (*P* = .09). Consequently, data from the right and left palatine tonsils were combined for further analyses, resulting in a total of 280 palatine tonsils evaluated.

#### Palatine tonsil contrast enhancement pattern, differentiation and fossa contents

**FIG 4:**
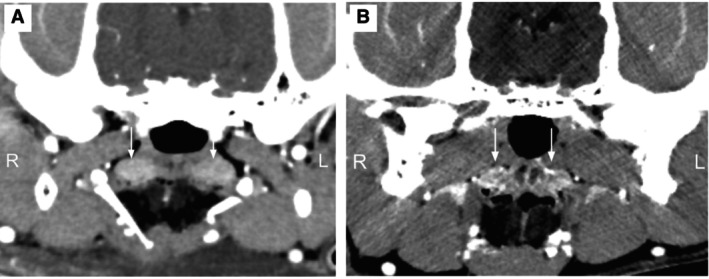
Contrast enhancement patterns of the palatine tonsils (arrows) shown on transverse plane images: (A) homogeneous contrast enhancement pattern; (B) heterogeneous contrast pattern.

**FIG 5:**
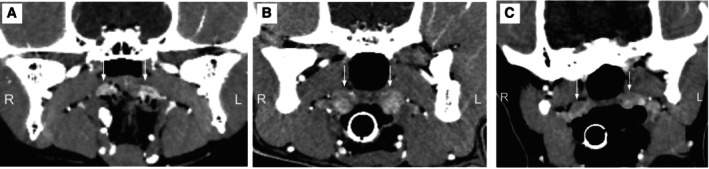
Differentiation of the palatine tonsils (arrows) to the surrounding soft tissues shown on transverse plane post‐contrast images: (A) good; (B) medium; (C) poor differentiation.

**FIG 6:**
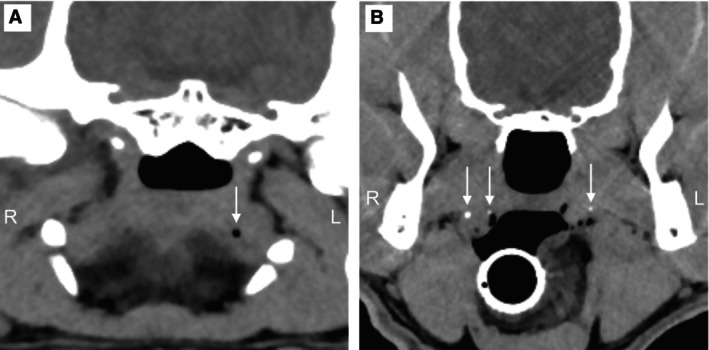
Transverse plane pre‐contrast images of the palatine tonsils showing: (A) one small focus of gas attenuation (arrow) in the left tonsillar fossa; (B) two small foci of mineral attenuation in the right tonsillar fossa, and one small focus of mineral attenuation in the left tonsillar fossa (arrowed).

All 280 palatine tonsils displayed soft tissue attenuation in pre‐ and post‐contrast imaging. The subjective observations performed on the palatine tonsils are summarised in Table 1. A homogeneous contrast enhancement pattern was observed in 65.7% of the palatine tonsils (Fig 4). No statistically significant differences were found between palatine tonsil contrast enhancement pattern and the level of post‐contrast enhancement in the vasculature (as measured in the carotid and jugular vessels; *P* = .04 and .58, respectively). A small majority (52.9%) of the palatine tonsils were poorly differentiated from adjacent soft tissues, while the remainder exhibited medium and good differentiation (Fig 5). No statistically significant differences were found between the degree of palatine tonsil differentiation from surrounding soft tissues and the level of post‐contrast enhancement in the vasculature (*P* = .12 and .03 for carotid and jugular vessels, respectively). Gas attenuation was identified in 86.1% tonsillar fossae, and mineral attenuation was identified in 7.9% tonsillar fossae (Fig 6).

**Table 1:** Summary of the subjective observations performed of 280 palatine tonsils, including contrast enhancement pattern, palatine tonsil differentiation from surrounding soft tissues on post‐contrast images, and contents in the palatine tonsillar fossae

*Palatine tonsil contrast enhancement pattern*	
Homogeneous	184 (65.7%)
Heterogeneous	96 (34.3%)
*Palatine tonsil differentiation from surrounding soft tissues on post‐contrast images*	
Good	42 (15.0%)
Medium	90 (32.1%)
Poor	148 (52.9%)
*Contents in the palatine tonsillar fossae*	
Gas	241 (86.1%)
Mineral	22 (7.9%)

#### Palatine tonsil differentiation relationship with brachycephalic conformation

No statistically significant associations were found between brachycephalic conformation (brachycephalic *vs*. normocephalic groups) and palatine tonsil differentiation from surrounding soft tissues (poor, medium and good differentiation) with *P* = .23.

#### Palatine tonsil attenuation

Palatine tonsil pre‐ and post‐contrast attenuation means did not follow a normal distribution. The increase in median attenuation mean from 50.8 HU pre‐contrast to 89.4 HU post‐contrast was statistically significant (*P* < .001). These results are summarised in Table 2. Statistically significant positive correlations were observed between palatine tonsil post‐contrast attenuation and the level of post‐contrast enhancement in the vasculature. Specifically, higher palatine tonsil post‐contrast attenuation means were seen with higher vascular (carotid and jugular) contrast (Spearman's rho = 0.34 and 0.44, respectively, both with *P* < .001).

**Table 2:** Pre‐ and post‐contrast mean attenuation (HU) median and range of 280 palatine tonsils

Pre‐contrast mean attenuation, median (range) (HU)	50.8 (22.6 to 82.1)
Post‐contrast mean attenuation, median (range) (HU)	89.4 (49.7 to 128.0)

#### Palatine tonsil size

Objective planar dimension measurements were performed on the 132 palatine tonsils exhibiting medium and good differentiation to surrounding soft tissues on post‐contrast images. The findings are summarised in Table 3. Palatine tonsil width, height and length all followed a normal distribution.

**Table 3:** Width, height and length (mm) mean, standard deviation (SD) and range of 132 palatine tonsils

Width mean (SD) (mm)	6.9 (2.2)
(range)	(2.2 to 13.2)
Height mean (SD) (mm)	6.5 (2.0)
(range)	(2.6 to 12.8)
Length mean (SD) (mm)	19.1 (4.5)
(range)	(7.4 to 32.9)

#### Palatine tonsil size relationship with age

A statistically significant weak negative correlation was found between age and palatine tonsil dimensions for the 132 palatine tonsils measured: width (Spearman's rho = −0.24, *P* = .006), height (Spearman's rho = −0.50, *P* < .001) and length (Spearman's rho = −0.25, *P* = .003).

#### Palatine tonsil size relationship with bodyweight

**FIG 7:**
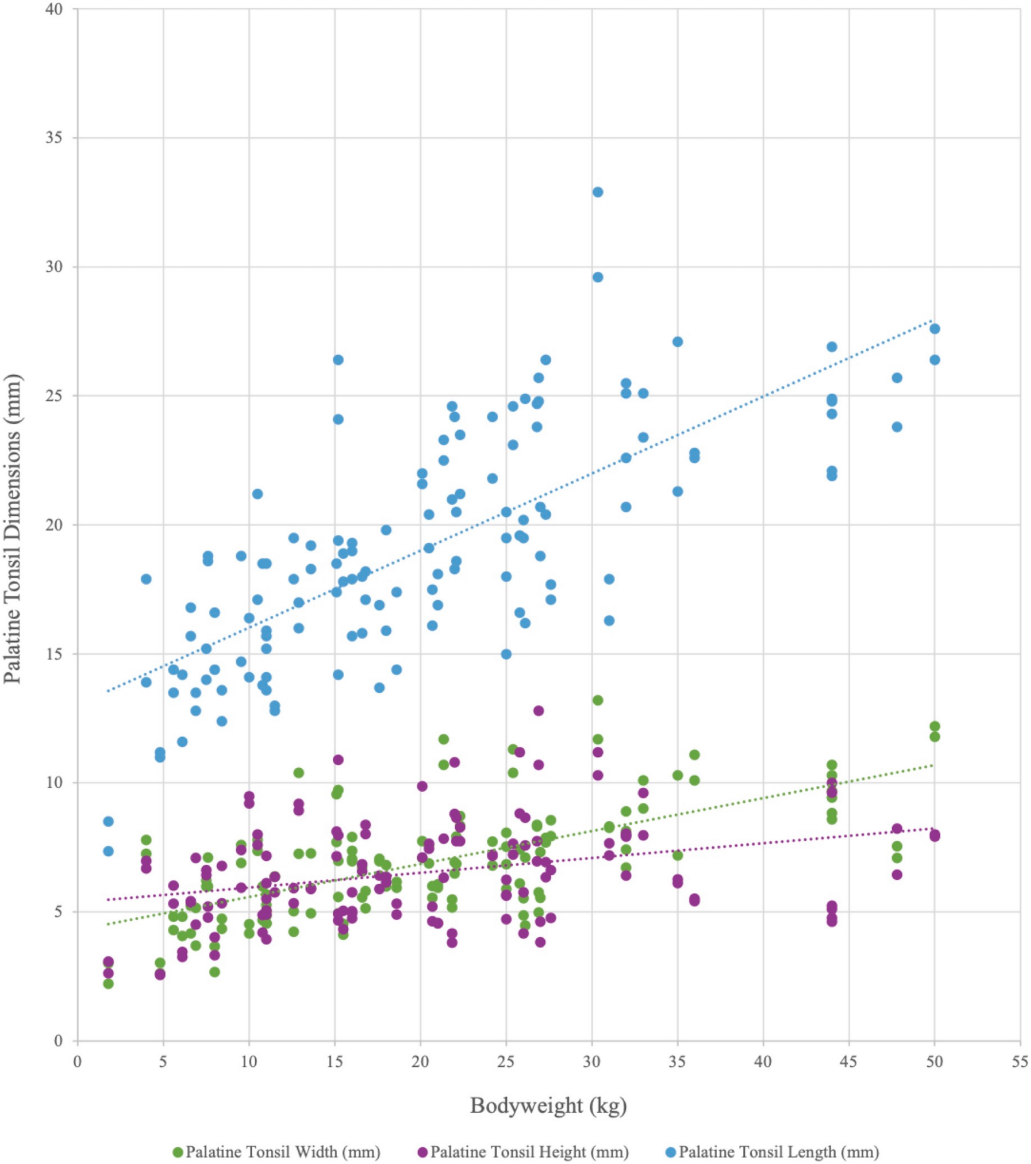
Scatterplot of bodyweight (kg) *versus* palatine tonsil width, height and length (mm) measured for 132 palatine tonsils. Statistically significant weak positive correlations (*P*‐values < .001) were found between bodyweight and each of the palatine tonsil dimension measurements.

Shown in the scatterplot in Fig 7, a statistically significant weak positive correlation was found between bodyweight and palatine tonsil dimensions for the 132 palatine tonsils measured: width (Spearman's rho=0.65), height (Spearman's rho=0.34) and length (Spearman's rho=0.76), with *P* < .001 for all dimensions.

Palatine tonsil width, height and length were normally distributed for each of the three bodyweight categories (≤10.0 kg, 10.1 to 25.0 kg and >25.0 kg), with measurements summarised in Table 4. The increase in palatine tonsil size with each ascending bodyweight category was significant (*P* < .001 for all dimensions).

**Table 4:** Summary of palatine tonsil width, height and length (mm) mean, standard deviation (SD) and range in three bodyweight categories (≤10.0 kg, 10.1 to 25.0 kg and >25.0 kg)

	≤10.0 kg (*n* = 26)	10.1 to 25.0 kg (*n* = 64)	>25.0 kg (*n* = 42)
Width mean (SD) (mm)	4.9 (1.6)	6.7 (1.6)	8.5 (2.1)
(range)	(2.2 to 7.8)	(4.1 to 11.7)	(4.5 to 13.2)
Height mean (SD) (mm)	5.4 (1.9)	6.4 (1.7)	7.4 (2.1)
(range)	(2.6 to 9.5)	(3.8 to 10.9)	(3.8 to 12.8)
Length mean (SD) (mm)	14.2 (2.9)	18.5 (3.1)	23.0 (3.7)
(range)	(7.4 to 18.8)	(12.8 to 26.4)	(16.2 to 32.9)

Statistically significant increases in palatine tonsil size measurements for each ascending bodyweight category were found

#### Palatine tonsil size relationship with brachycephalic conformation

Palatine tonsil width, height and length, for palatine tonsils exhibiting medium and good differentiation to surrounding soft tissues on post‐contrast images, were normally distributed for brachycephalic (*n* = 24) and normocephalic (*n* = 108) dogs, with measurements for both groups summarised in Table 5. No statistically significant differences were found between the two groups for palatine tonsil width, height or length (*P* = .530, .360 and .032, respectively).

**Table 5:** Summary of palatine tonsil width, height and length (mm) mean and standard deviation (SD) in 24 palatine tonsils from brachycephalic breeds and 108 palatine tonsils from normocephalic breeds

	Brachycephalic (*n* = 24)	Normocephalic (*n* = 108)
Width mean (SD) (mm)	7.1 (1.3)	6.7 (1.4)
Height mean (SD) (mm)	6.9 (1.7)	6.2 (2.0)
Length mean (SD) (mm)	17.3 (2.6)	19.5 (4.8)

No statistically significant differences were found between palatine tonsil sizes in the brachycephalic group compared to the normocephalic group

#### Palatine tonsil size relationship with concurrent regional disease

Palatine tonsil width, height and length, for palatine tonsils exhibiting medium and good differentiation to surrounding soft tissues on post‐contrast images, were normally distributed for dogs with reported concurrent head, neck or dental disease (*n* = 96), as well as dogs with no reported disease (*n* = 36). No statistically significant differences were found between the two groups for palatine tonsil width, height or length (*P* = .793, .564 and .769, respectively).

## DISCUSSION

This study offers a comprehensive analysis of the CT features of presumed normal canine palatine tonsils, providing valuable insights into their appearance and characteristics. Consistent with prior research, lymphoid tissue in the tonsils displayed soft tissue attenuation on CT scans, with all tonsils showing contrast medium uptake. This finding aligns with the known contrast‐enhancing properties of lymphoid tissues in the dog, as well as previously described enhancement patterns observed in the oropharynx (Forrest & Saunders, 2011; Kneissl & Probst, 2007; Milovancev et al., 2017). This study found that 52.9% of the tonsils were poorly differentiated from surrounding soft tissues on post‐contrast images, in agreement with previous reports on isoattenuation of tonsils (Murakami et al., 2018). Proactively adjusting the window width and level on post‐contrast CT studies was useful to differentiate structures with similar attenuation values, as recommended in the literature (Saunders & Schwarz, 2011). Rewindowing was consistently applied by reviewers as needed, but it did not affect the evaluation of the structures, as confirmed during the consensus review. While the current study found a positive correlation between post‐contrast tonsil attenuation and the level of vascular enhancement, the lack of a statistically significant difference between tonsillar contrast enhancement pattern and the level of post‐contrast vascular enhancement suggests that post‐contrast tonsillar enhancement may not be solely influenced by the timing of contrast injection. Likewise, vascular enhancement did not differ significantly across tonsils with poor, medium or good differentiation from surrounding tissues, implying that other factors may influence the appearance of these structures on CT. These observations are consistent with the principle that contrast enhancement occurs both through blood flow and vascular permeability (Pollard & Puchalski, 2011).

Tonsils exhibited homogeneous contrast enhancement in 65.7% of cases, while 34.3% showed heterogeneous patterns, which may reflect normal physiological variation or underlying pathology. This is consistent with MRI findings, where 57% of canine palatine tonsils showed heterogeneous signal intensity (Ruiz‐Drebing et al., 2019). The current study also found no statistically significant difference in the appearance of right and left palatine tonsils, as expected anatomically (Evans & Lahunta, 2013). The presence of gas attenuation in 86.1% of the tonsillar fossae, which had been previously proposed as a distinguishing feature of canine palatine tonsils (Forrest & Saunders, 2011), was also observed. This reinforces the idea that gas attenuation is a reliable marker for identifying the tonsils on CT scans. Mineral attenuation was identified in only 7.9% of fossae. To the authors' knowledge, this is the first report of mineral content in canine tonsillar fossae, as such findings have not been previously documented in dogs, despite being frequently found in humans (Takahashi et al., 2014).

This study also explored the relationship between palatine tonsil size and various factors, such as age and bodyweight. A weak negative correlation was observed between palatine tonsil size and age, supporting previous reports that lymphoid tissue, including palatine tonsils, undergoes age‐related regression in dogs (Burns et al., 2008; Krol & O'Brien, 2012; Ruppel et al., 2019). Additionally, a significant positive correlation was found between bodyweight and tonsil size, with larger dogs having larger palatine tonsils, as expected due to physiological scaling. The study further categorised dogs by bodyweight (≤10.0 kg, 10.1 to 25.0 kg and >25.0 kg), with larger tonsil sizes observed in heavier dogs. This information may be valuable for clinicians, as it may influence the surgical approach for tonsillectomy, where anatomical differences in tonsil size across bodyweight categories could affect the procedure. Various complications, such as bleeding, have been reported following tonsillectomy in dogs; however, further research is needed to explore whether complication rates are associated with bodyweight (Belch et al., 2017; Turkki et al., 2022). Interestingly, no statistically significant differences in tonsillar size were found between brachycephalic and normocephalic dogs, aligning with MRI findings (Köhler et al., 2021). Additionally, no statistically significant differences in tonsil size were found in dogs with concurrent regional disease, which is expected given that the palatine tonsils lack afferent lymphatics (Belz & Heath, 1995; Evans & Lahunta, 2013). This contrasts with the established association between enlarged lymph nodes and dental disease in dogs (Belotta et al., 2022; Harvey, 2022). Overall, these findings suggest that factors such as bodyweight and age play a more crucial role in determining palatine tonsil size, rather than head conformation or concurrent regional disease.

A limitation of this study includes its retrospective design, which led to variations in imaging protocols, such as slice thickness and image acquisition timing. These inconsistencies may have introduced variations in attenuation and size measurements. Additionally, size measurements were only made for tonsils with medium or good differentiation from surrounding tissues, leaving out poorly differentiated tonsils, which may have impacted the representativeness of size assessments and generalisability of the results. As no statistically significant differences were detected between right and left tonsils, each tonsil was subsequently treated as an individual data point. However, because tonsils are paired structures and therefore not strictly independent, this represents a limitation of the study's statistical analyses. Furthermore, no histopathological confirmation of the tonsils' normalcy was conducted, so these findings should be viewed as reference data rather than definitive criteria for normal tonsillar characteristics. Due to the retrospective nature of this study and the lack of histopathological or clinical examination of this area, it is possible that the identified mineralisation or gas was located within the tonsillar parenchyma rather than the fossae; however, this is considered less likely. Intraobserver and interobserver variability were not assessed, although a consensus review was performed by a board‐certified radiologist. While the study involved a broad range of ages and body weights, it predominantly included medium to small‐breed dogs. Therefore, these findings may not fully apply to larger breeds or dogs with different anatomical characteristics. Further studies should examine these features in larger dogs, as well as a larger cohort of brachycephalic breeds, where tonsillar abnormalities and differences in upper airway structures may occur due to breed‐specific anatomy (Ekenstedt et al., 2020; Fasanella et al., 2011). Prospective studies that include histopathological confirmation and standardised imaging protocols would provide more reliable data, enhancing the clinical utility of these findings.

In conclusion, this study significantly enhances the understanding of the CT characteristics of canine palatine tonsils, providing a valuable reference for veterinarians when assessing these structures. The data gathered here may help clinicians distinguish between normal and abnormal tonsils, aiding in decision‐making for diagnostic and therapeutic interventions. The information from this study may also help support surgical planning for tonsillectomies. Future research, particularly studies involving histopathological correlation and alternative imaging modalities, would refine these findings, improving the diagnostic accuracy and clinical management of tonsillar disease in dogs.

## Author contributions

**N. Leong:** Conceptualization; investigation; writing – original draft; methodology; validation; writing – review and editing; formal analysis; data curation; project administration; visualization. **R. Drees:** Conceptualization; investigation; methodology; validation; formal analysis; supervision; data curation; project administration; visualization; writing – review and editing; writing – original draft. **H. Dirrig:** Conceptualization; investigation; methodology; validation; supervision; data curation; project administration; visualization; writing – review and editing; writing – original draft.

## Conflict of interest

None of the authors have a conflict of interest to disclose.

## Supporting information


Appendix Table S1.

## Data Availability

The data that support the findings of this study are available from the corresponding author upon reasonable request.
