# Supplementary material for: Computed tomographic features of presumed normal palatine tonsils in 140 dogs
Source: J Small Anim Pract. 2026 Apr 12;67(8):739–48. doi: 10.1111/jsap.70132 (PMC13448747; doi:10.1111/jsap.70132)
Supplement: Supplementary file 1 [file JSAP-67-739-s001.docx]

Appendix Table S1: 50 different breeds and mixed breed represented by the 140 dogs included in the study.

| Breed | Number of dogs |
| --- | --- |
| Cross Breed | 30 |
| Cocker spaniel | 9 |
| Labrador retriever | 9 |
| French Bulldog † | 8 |
| Springer spaniel | 8 |
| Golden retriever | 5 |
| Staffordshire Bull Terrier † | 5 |
| Bulldog † | 4 |
| Border Collie | 3 |
| Cavalier King Charles Spaniel † | 3 |
| Dachshund | 3 |
| German shepherd | 3 |
| Miniature schnauzer | 3 |
| Yorkshire terrier | 3 |
| Irish Setter | 2 |
| Jack Russell Terrier | 2 |
| Maltese terrier | 2 |
| Miniature dachshund | 2 |
| Pug † | 2 |
| Tibetan Terrier | 2 |
| Whippet | 1 |
| American Staffordshire Terrier | 1 |
| Australian Shepherd | 1 |
| Bernese Mountain Dog | 1 |
| Border Terrier | 1 |
| Boxer | 1 |
| Bull Terrier | 1 |
| Chihuahua † | 1 |
| Dalmatian | 1 |
| Deer Hound | 1 |
| German Long-Haired Pointer | 1 |
| Grand Basset Griffon Vendéen | 1 |
| Hungarian Vizsla | 1 |
| Irish Red & White Setter | 1 |
| Italian Greyhound | 1 |
| Lakeland Terrier | 1 |
| Lhasa Apso † | 1 |
| Norfolk Terrier | 1 |
| Parson Russell Terrier | 1 |
| Patterdale Terrier | 1 |
| Pomeranian | 1 |
| Portuguese Pointer | 1 |
| Rottweiler | 1 |
| Samoyed | 1 |
| Scottish Terrier | 1 |
| Shih Tzu † | 1 |
| Soft-Coated Wheaton Terrier | 1 |
| Standard poodle | 1 |
| Tibetan Spaniel † | 1 |
| Welsh Springer Spaniel | 1 |

^†^ Breed is included in the brachycephalic group.
